# Frederick Weatherly

**Published:** 1910-09

**Authors:** 


### FREDERICK WEATHERLY, M.R.C.S. Eng., L.S.A., J.P.

One of the oldest practitioners of the West-country passed away on August 11th, and general was the regret among the number who knew him when the sad, though not unexpected, announcement was made that Frederick Weatherly, of Portishead, had gone over to the majority.

Educated at Great Ealing School, with Professor Huxley, Cardinal Newman, Sir Henry Rawlinson, and many other notables as his school companions, he entered when almost a boy at St. Bartholomew's Hospital. Many are the stories he has told me of his hard life in the slums round that well-known hospital, and great was the practical knowledge he there gained, inculcating to a great degree those faculties of keen observation and quick perception which for years stood him in such good stead.

To-day instruments of precision are ever growing more numerous, more exact, more trustworthy. The thermometer, the ophthalmoscope and laryngoscope were unknown in his day, while bacteriology had not been dreamt of. It was upon the organs of sense that such a man as he had to depend—sight, hearing, and touch had to take the place of the present diagnostic instrumental armamentaria.

I often look back to those days when as his apprentice I noted his wonderful and quick perceptive and observing powers, and men like Lyons, Symonds, Budd, Prichard, and Fox, whom he often called into consultation, always had a word of praise for his wise discernment. Work he loved, and his kindly voice, his unsparing efforts to relieve suffering and fight death, made him widely popular and universally respected.

He came to Portishead in 1846, then little more than a pretty fishing village, and there he worked for forty years. It was his proud boast that for thirty-six years he never was away from his work for twenty-four hours. How many country practitioners can say this ?

The year of his arrival saw him married to my dear mother, the daughter of the late Alexander Ford, of Bristol, and sister of the late James Ford, of Wraxall Court, who for so many years was leader of the Conservative party in Bristol.

He retired from active medical practice in 1886, though for some few years before this date he had relinquished a great deal of his medical work, and had determined to devote himself to public service. How well he carried that determination out is common knowledge in the West of England.

He was Chairman of the Bedminster Board of Guardians, afterwards, on the extension of the Bristol boundaries, changed to Long Ashton Board ; he was the first Chairman of the Long Ashton Rural District Council ; he was a Justice of the Peace for Somerset and County Councillor, Chairman of Bedminster and Clevedon Technical Education Committee, Commissioner of Taxes, and for many years churchwarden of the parish church.

If it had not been that in his early days of practice he had the good fortune to take under his care a gentleman who lived with him for years, he could not have given five of his sons a University education, though he was always proud of the fact that they too contributed to their educational expenses by scholarships and exhibitions. This gentleman had been a schoolfellow of Thackeray at Charterhouse, a great friend of Gladstone at Christ Church, and had travelled with Sir Walter Scott. Through him my father became known to Gladstone and many are the letters and post cards we have received from that wonderful man.

Twelve years ago, on the 15th of August, 1898, he lost my dear mother, his helpmate for so many years, and it was a touching coincidence that on the same date of this year he was laid to rest beside her.

We were thirteen in family—eight sons and five daughters, of whom seven sons and one daughter are alive to-day.

Some three years ago the past and present members of the

Long Ashton Board of Guardians, with other friends to the number of 165, marked their appreciation of his work as their Chairman for twenty-five years and of his unique service to the district in which he lived by the presentation of a handsome silver service, and the speeches made on that memorable day were full of testimony of all that his work, his tact, his energy, and his keen intellect had done for the good and for the betterment of those amongst whom he dwelt.

On Monday, August 15th, he was carried to his last resting-place on the shoulders of men whom he had assisted into the world, and by whose special request the simple and impressive funeral was arranged. It was a day to be ever remembered by those who took part in it, and proved, if proof were needed, the love and respect of those who had known him in the spring, summer, and autumn of his life.

